# Anti-Leucine-Rich Glioma-Inactivated 1 (Anti-LGI 1) Limbic Encephalitis and New-Onset Psychosis: A Case Report

**DOI:** 10.7759/cureus.36223

**Published:** 2023-03-16

**Authors:** António Alho, Nuria F Santos, Rita Felício, Carlos J Vieira

**Affiliations:** 1 Departamento de Psiquiatria e Saúde Mental, Hospital Distrital de Santarém, Santarém, PRT; 2 Serviço de Psiquiatria, Hospital Prof. Dr. Fernando da Fonseca, Amadora, PRT

**Keywords:** liaison psychiatry, psychosis, anti-leucine-rich glioma-inactivated 1 limbic encephalitis, limbic encephalitis, autoimmune encephalitis

## Abstract

Anti-leucine-rich glioma-inactivated 1 limbic encephalitis (Anti-LGI 1 LE) is a subtype of autoimmune encephalitis (AE) and the most common cause of limbic encephalitis (LE). Clinically, it can have an acute to sub-acute onset of confusion and cognitive impairment, facial-brachial dystonic seizures (FDBS), and psychiatric disturbances. The clinical manifestations are varied, and its diagnosis requires high clinical suspicion to avoid delay in the treatment. When patients manifest mostly psychiatric symptoms, the disease may not be immediately recognized. We aim to report a case of Anti-LGI 1 LE in which the patient presented acute psychotic symptoms and was initially diagnosed with unspecified psychosis. We present a case of a patient with sub-acute behavioral changes, short-term memory loss, and insomnia who was brought to the emergency department after a sudden episode of disorganized behavior and speech. On medical examination, the patient presented persecutory delusions and indirect signs of auditory hallucinations. An initial diagnosis of unspecified psychosis was performed. Investigations revealed right temporal epileptiform activity in the electroencephalogram (EEG), abnormal bilateral hyperintensities in the temporal lobes in the brain magnetic resonance imaging (MRI), and a positive titer of anti-LGI 1 antibodies (Abs) in serum and cerebrospinal fluid (CSF), leading to a diagnosis of anti-LGI 1 LE. The patient was treated with intravenous (IV) steroids and immunoglobulin and later with IV rituximab. In patients that predominantly present with psychotic and cognitive disorders the diagnosis of anti-LGI 1 LE can be delayed predisposing them to a poorer prognosis (permanent cognitive impairment - especially short-term memory loss - and persistent seizures). It is necessary to be aware of this diagnosis when evaluating acute to sub-acute psychiatric illness developing with cognitive impairment (specially with memory loss) to avoid diagnosis delays and long-term sequelae.

## Introduction

Autoimmune encephalitis (AE) is a type of neurological autoimmune disease caused by the presence of autoantibodies (Abs) on the neuronal surface or intracellular antigens. Different subtypes of AE are distinguished by their specific Abs [1]. Among them, anti-leucine-rich glioma-inactivated 1 limbic encephalitis (anti-LGI 1 LE), is a treatable etiology of AE. Anti-LGI 1 Abs were found in 2010, which is thought to be the second most common cause of AE following anti-N-methyl-D-aspartate receptor (NMDAR) encephalitis and the most common cause of LE [2]. Anti-LGI 1 LE, a subtype of AE, is characterized by an antibody-mediated inflammation of the limbic region [3].

Anti-LGI 1 LE has an incidence of around 0.83 per 100 million [4] and typically evolves and predominantly affects middle-aged and elderly males over 50 years old [1]. A tumor association was found in up to 11% of the cases, with thymomas and lung carcinomas being the most common types [5]. Clinically, it can have an acute to subacute onset of confusion and cognitive impairment, facial-brachial dystonic seizures (FDBS), convulsions, psychiatric disturbances, and refractory hyponatremia [2,6]. Regarding the treatment, it usually shows a good response to immune-mediated treatment [7]. Anti-LGI 1 LE can be diagnosed through clinical features, magnetic resonance imaging (MRI), serum or cerebrospinal fluid (CSF) tests and electroencephalogram (EEG). The gold standard for diagnosis is a positive LGI 1 Ab in serum or CSF [8]. Because anti-LGI 1 LE can present with a broad spectrum of clinical manifestations high clinical suspicion is needed in order to diagnose it and avoid treatment delays. When patients manifest mostly psychiatric symptoms, the disease may not be immediately recognized. Because of the rarity of this condition, these patients pose a management challenge [7]. We aim to report a case of anti-LGI 1 LE, in which the patient presented acute psychotic symptoms and was initially diagnosed with unspecified psychosis.

## Case presentation

A 26-year-old female patient was brought to the emergency department (ED) by her mother due to behavioral changes over the last month with a sudden episode of disorganized behavior and speech the previous night. She was found by her mother screaming at one window of their house that someone wanted to hurt her and that she wanted someone to call the police. Earlier that day, she called her mother to tell her she was afraid that someone was trying to hurt her. She also said that she was hearing voices that threatened her. The patient’s mother mentioned that during the previous month, she exhibited subtle alterations, namely a decline of energy to perform daily tasks, social withdrawal, disorganized and slurred speech, insomnia, decreased appetite, and short-term memory impairment. The patient said that she was brought by her mother to the ED because she was caught screaming at the window for help, after feeling a shiver, which could mean someone was trying to hurt her, and that she did it because she felt a shiver before that which could mean that someone was trying to hurt her.

The patient denied a known history of medical problems or psychiatric illness and did not have any drinking or smoking habits. However, after consulting the National Health Data Platform, two records of her family’s doctor were found, in 2018, one referring to disorientation in time and space, insomnia, and unspecified memory complaints, in April, and the other reporting that the patient seemed to have depressive humor which led to a prescription of escitalopram, later in November. There are no medical records after that, and her mother did not mention any behavioral changes until the month before she was brought to the ED. She wasn’t currently taking any medication and did not use illicit substances. Her family psychiatric history was unremarkable.

On the mental status examination (MSE) she was conscious and hypervigilant. She was oriented to herself but it was impossible to assess the remaining components of orientation, because of the patient's refusal to answer to questions to assess this parameter; in general, she was moderately collaborative. Her facies were predominantly interrogative and perplexed in some moments. The patient kept her ability of focused attention, but her capacity for sustained attention was frankly diminished. The debt of speech was decreased, and it was vague and uninformative, with tangential answers. She had some periods of emission of hisses and clicks while staring at the interviewer, although she could not explain the reason why and made faces and laughers denoting vocal and motor stereotypies. The patient presented retardation of thinking and loosening of associations. It was impossible to assess the content of the patient's thinking because of her lack of cooperation to assess it. She presented indirect signs of auditory hallucinations like soliloquies, unmotivated laughs, and interruptions of speech to answer external sources. There were possible tactile hallucinations and/or passivity phenomena. She had initial insomnia and she lacked insight. The patient did not have any loss of strength in her limbs, rigidity, catalepsy, or wax flexibility, and neither did she have automatic obedience, echolalia, echopraxia, or negativism. She seemed to present ambitendency, although she could carry out simple instructions with latency time.

Her first complete blood count, renal and liver function tests, thyroid function tests, C-reactive protein, anti-*Treponema* *pallidum* antibody, serologic screening for hepatitis and human immunodeficiency virus, urine analysis, toxic screening (urine and blood test), COVID-19 polymerase chain reaction (PCR) test and head computed tomography (CT) were all unremarkable.

The patient was admitted voluntarily (despite her lack of insight into the disease she accepted to do the necessary investigation and treatment), waiting for a vacancy in the Psychiatry Department in the ER Observation Room (OR) and she was given olanzapine 10mg at night. A preliminary diagnosis of Schizophrenia or other primary psychotic disorders (6A2Z according to the International Classification of Diseases, 11th revision - ICD 11) was given.

Because of the difficulty to obtain additional data in the first psychiatric evaluation, a second interview was performed on the following day. At that moment the patient tried to normalize the reported behavioral changes and to justify them with fatigue resulting from her excess work. She said she screamed out the window because she woke up thinking that there was a fire at her place, which she thought was real and because of that she also started to knock at all doors to wake her family. She stated that her phone was being cloned because Google sent her some messages (“Are you ok?”) and she also believed that someone was watching her messages and taking her some photographs because she saw a flash at her phone once. The patient also said that when she was on the street people were performing some tests on her to better understand “how Portuguese, Brazilians, and people of all races relate to each other” and that she felt invaded by these tests. She also noted once that someone on the television tried to send her an indirect message, but later she realized it was not what she was thinking.

**Figure 1:**
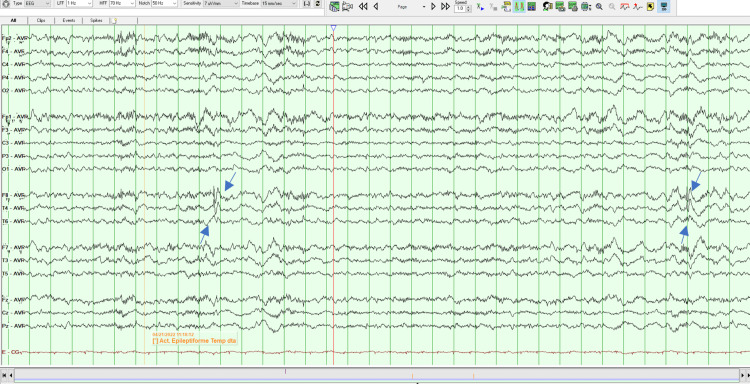
Electroencephalogram - Frame 1 Right temporal epileptiform activity, as highlighted with arrows

**Figure 2:**
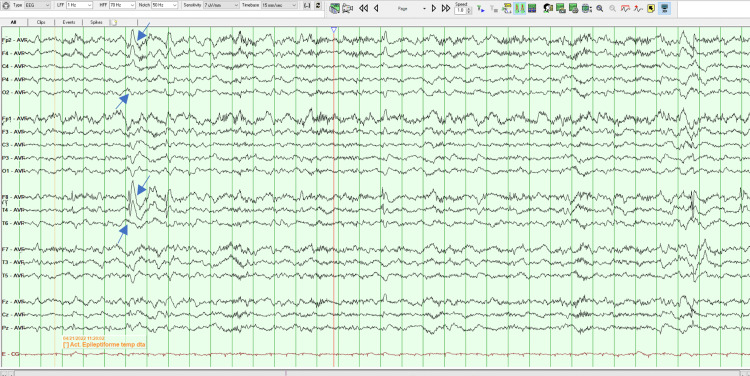
Electroencephalogram - Frame 2 Right temporal epileptiform activity, as highlighted with arrows

**Figure 3:**
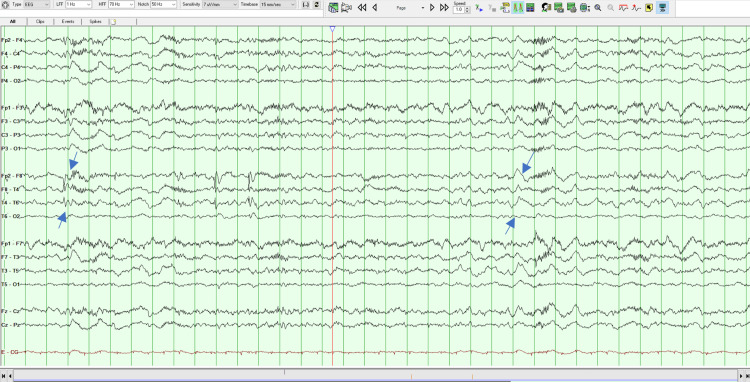
Electroencephalogram - Frame 3 Right temporal epileptiform activity, as highlighted with arrows

**Figure 4:**
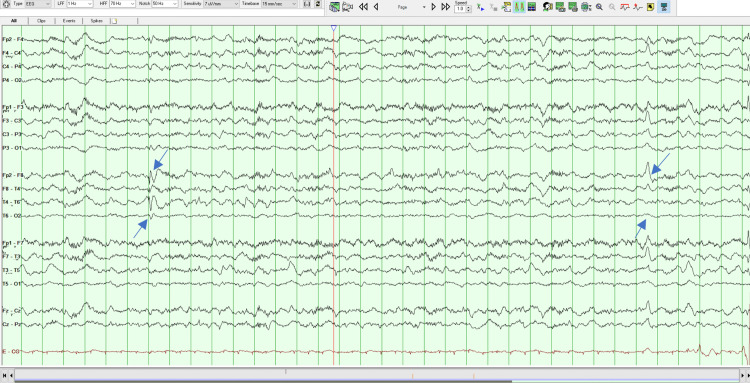
Electroencephalogram - Frame 4 Right temporal epileptiform activity, as highlighted with arrows

On her second MSE, the patient was oriented but less collaborative, asking repeatedly if she had to answer again to all those questions. Her speech remained vague and uninformative but without the vocal and motor changes noted the day before. She had persecutory delusions, but she did not have any more indirect signs of auditory hallucinations. The remaining of the MSE was similar to the previous evaluation. Later that day, an EEG was done which revealed the existence of right temporal epileptiform activity, as can be seen in Figures 1-4. A neurology evaluation was then requested.

 

**Figure 5:**
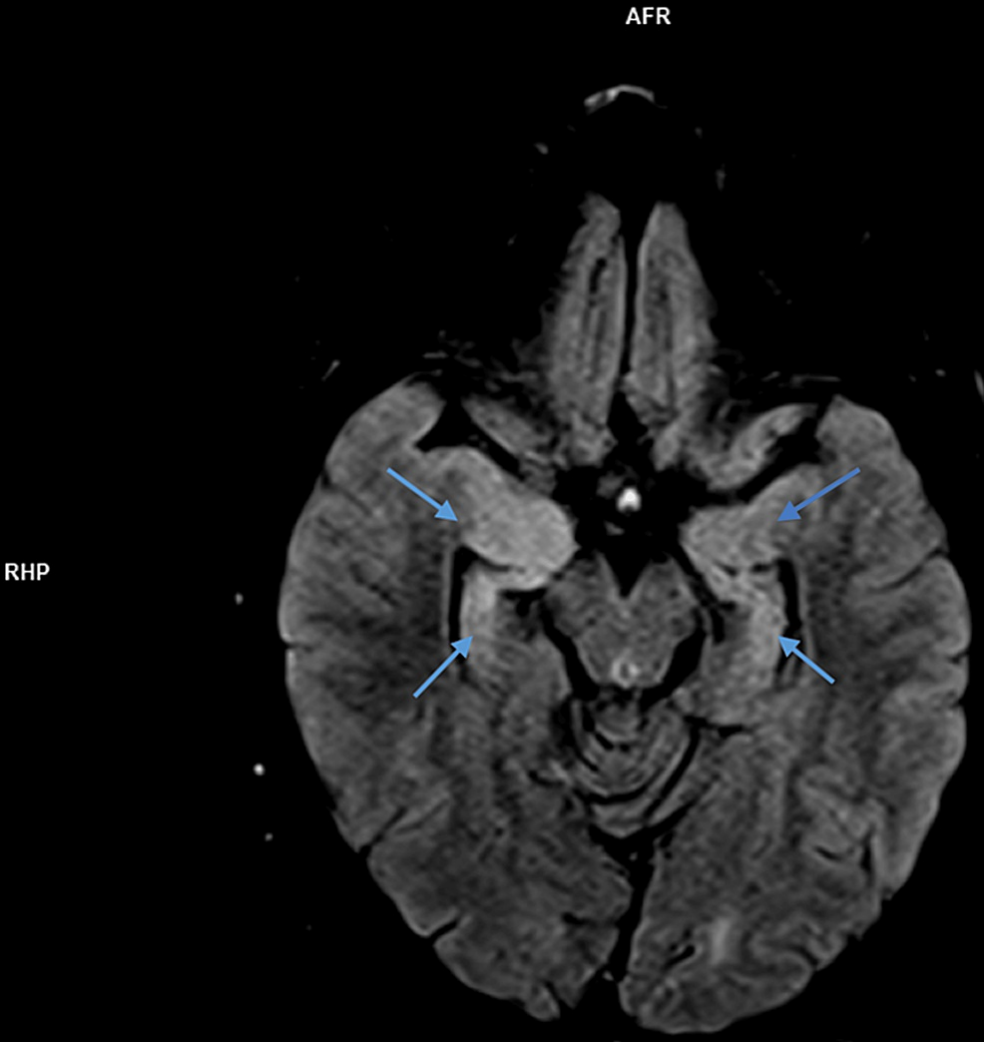
Brain MRI showing bilateral hyperintensities in the temporal lobes

Her full neurological evaluation was unremarkable, except for a slurred speech and a slight tremor in the finger-to-nose test. A lumbar puncture was then performed and the CSF cytologic and biochemical studies were all negative. Due to the patient's symptoms, EEG results, and after excluding an infectious etiology, a diagnosis of AE was assumed, and the patient was admitted to the Neurology ward. A five-day treatment with IV methylprednisolone 1,000mg a day, followed by sequential oral tapering with prednisolone (starting with 60mg a day) was initiated. In addition, an antiepileptic drug (levetiracetam 1,000mg twice a day) was started and the antipsychotic (olanzapine) was increased to 15mg a day. The patient's brain MRI indicated abnormal bilateral hyperintensities in the temporal lobes, which can be seen in Figure 5.

An extensive serum and CSF evaluation for other viral, bacterial, and fungal etiologies were negative, except for the hepatitis B surface and core antibodies whose results indicated a previous hepatitis B infection and were not contributive to the current case. The auto-immunity panel was positive only for anti-LGI 1 Ab both in serum (1:20) and CSF (1:20) and for serum voltage-gated potassium-channel antibodies (VGKC-Ab) whose titer was also elevated, at 424 pmol/liter (normal range: under 72 pmol/liter).

A diagnosis of anti-LGI 1 LE was made based on the neuropsychiatric symptoms, EEG and MRI findings, and the presence of anti-LGI 1-Ab and VGKC-Ab. Further work-up to rule out occult malignancy was done that included a CT of the chest, abdomen, and pelvis, an ultrasound of the pelvis and thyroid, mammography, a whole-body positron emission tomography (PET), and serum tumor markers which were all negative.

Another EEG was performed on the sixth day after admission which revealed the absence of epileptiform activity. Despite the absence of any neurological symptoms, the patient maintained persecutory delusions, saying that she continued to be watched, that her phone had been cloned, and that someone was withdrawing money from her bank account. Because of the presence of psychiatric symptoms as the predominant manifestation of the disease, a liaison psychiatry evaluation was requested. During this interview, it was indeed confirmed that the patient continued to present persecutory and self-reference delusions, although its impact seemed to be less significant than before. It was decided to keep an expectant attitude without proceeding to therapeutic changes.

One week later, she was again evaluated by liaison psychiatry, based on the persistence of delusions, to evaluate the necessity to proceed with the administration of additional immunotherapy. Throughout this reassessment, she denied being worried about the previous delusional complaints, although she added that she had some concerns about her professional future because she was afraid that her mother and some hospital workers could give future employers information about her health condition and with that jeopardize possible future job opportunities. She justified this possibility with the belief that everyone at the hospital talked about all the patients and she was no exception. Furthermore, she referred to multiple nocturnal awakenings with difficulty to resume sleep. It was then concluded that despite the apparent initial reduction in the impact of the delusional activity, not only it had not decreased, but had been extended with the addition of new data to the previous delusions. Having all of this in mind, it was considered that the psychotic symptoms had not improved, on the contrary, they got worse, which justified additional immunotherapy. The olanzapine dose was increased to 20mg a day.

A regimen of five days of IV immunoglobulin was then started (a total of 115 g) and a slight improvement was observed. The patient began to present some insight about her condition, assuming the need to remain admitted and to comply with therapy, but because of the endurance of her unjustified concerns about the possible third-party interference in her bank account it was decided to start second-line immunotherapy with IV rituximab and the first administration (1 g) was carried out during her hospitalization, after prophylactic treatment for hepatitis B reactivation (tenofovir) because of her analytical results consistent with previous infection. This last treatment led to near-full remission of psychotic symptoms. A neuropsychological evaluation was done right before discharge in which the patient revealed a low intelligence quotient (70-79), a slight slowing down of information processing speed, a slight defect in the divided-attention span, and psychomotor retardation without the relevant commitment of memory processes and executive functions.

She was then discharged with levetiracetam 2,000 mg/day, olanzapine 20 mg/day, and a tapering dosage of oral steroid (prednisolone), being the subsequent administrations of IV rituximab scheduled for the day hospital. Follow-up appointments with Neurology and Psychiatry were programmed. Despite the multiple notices, the patient repeatedly missed her appointments, showing up afterward at the hospital to reschedule. When she finally attended her Neurology consultation, she had stopped all of her medication (it was impossible to confirm for how long), denying any complaint. However, her behavior was again suggestive of some degree of psychotic activity. Levetiracetam 1,000 mg/day and olanzapine 15 mg/day were reinstituted. One month after, the patient remained stable, without psychotic symptoms, normal behavioral activity, regular sleep pattern, and complying with medication. She had also returned to work. An EEG and analytical control studies were pending for the following Neurology visit.

## Discussion

AE accounts for 10%-20% of cases of encephalitis, with anti-NMDAR encephalitis being the most common, accounting for about 80% of AE encephalitis, followed by anti-LGI 1 encephalitis. Anti-LGI 1 Ab-associated encephalitis mainly involves the limbic system and is called autoimmune LE. LE is an AE involving the limbic system, including the medial temporal lobe, amygdala, hippocampus, cingulated cortex, and insular lobe [2]. Together, these structures play a crucial role in one’s emotions, learning, memory, and motivation. LE is classically recognized as a paraneoplastic disease, associated most often with small-cell carcinoma of the lung, various testicular tumors, thymoma, breast cancer, and Hodgkin lymphoma. Nevertheless, it is now understood that LE can also manifest secondary to an autoimmune or post-infectious process [3].

LGI 1 is a functional component of the VGKC in limbic neurons. While the pathophysiology of LE is not completely understood, it is thought that the antibodies targeting LGI 1 disrupt the function of VGKC, leading to hyperexcitability of neurons found in the limbic system [3] and resulting in epileptic seizures. The antigen-antibody reaction in anti-LGI 1 LE leads to inflammatory changes in the hippocampus which eventually results in atrophy or sclerosis of the hippocampus and a permanent decline in cognitive function [1].

Ab production in LE was once thought to arise solely in the context of paraneoplastic disease; however, individual case reports in the last decade demonstrate anti-LGI 1 Ab in the CSF without an underlying malignancy. Thus, LE can arise from both paraneoplastic and independent autoimmune or post-infectious processes, the latter now clinically presenting more frequently in the current literature [3]. In our case, the presence of malignancy and infection were both excluded in the available performed studies. Anti-LGI 1 LE can be diagnosed through clinical features, MRI, serum or CSF tests, and EEG. The gold standard for diagnosis is a positive LGI 1 Ab in serum or CSF [8].

Clinically anti-LGI 1 LE is characterized by cognitive impairment, memory loss, FDBS, sleep disorders, psychiatric disturbances, convulsions, and hyponatremia [2-4], and is seen in older people (mainly in males) with an average age of onset around 63 years of age [4]. Cognitive impairment is the most common manifestation. Memory disorders, especially those affecting short-term memory, are the most prominent [2]. In addition to cognitive decline, seizures are another major clinical feature of anti-LGI 1 LE, with an incidence of 65% to 82%. FDBS is the characteristic seizure type that presents as short, frequent, and stereotyped dystonic movements of the face and the ipsilateral arm and/or leg, frequently preceding the onset of anti-LGI1 encephalitis [1]. Hyponatremia has also been found to be a characteristic finding, although non-specific and associated with many seizure disorders [3]. LGI 1 is expressed in the renal tubular system, which provides another potential mechanism for the dysregulation of the sodium balance [5]. Our patient was 26 years old, female, and presented with insomnia, short-term memory disturbances, and psychotic symptoms which were the most prominent and persistent features of the disease. In addition to being young our patient was female which makes this case even more exceptional. The patient never had FDBS, the characteristic seizure for anti-LGI 1 LE or epileptiform discharges. In the beginning, some stereotyped movements of the face were observed which disappeared the day after admission, but the remaining features of FDBS were absent.

MRI findings of anti-LGI 1 LE generally show abnormal FLAIR or T2 hyperintensity in the hippocampus and medial temporal lobe which may be unilateral or bilateral [4,7]. CSF findings may be inconspicuous or may show an increased protein concentration or a slight pleocytosis. EEG abnormalities are detected in 75% of patients with anti-LGI 1 LE, typically with frontotemporal slowing of background activity [7]. In a study of 2022 by Teng et al., the positive rate of LGI 1 Abs in CSF was 77.38% and the positive rate in serum was 96.83%, suggesting a higher sensitivity in diagnosing anti-LGI 1 LE. Our patient brain MRI indicated abnormal bilateral hyperintensities in the temporal lobes, being her CSF cytochemical study unremarkable. Her EEG demonstrated right temporal epileptiform activity and there was a positive title for anti-LGI 1 Ab both in serum (1:20) and CSF (1:20). The serum voltage-gated potassium-channel antibodies (VGKC-Ab) were also elevated. All the previous results combined with the clinical characteristics fulfilled the diagnosis of anti-LGI-LE.

Although there is no clear standardized treatment, immunotherapy, including first-line drugs - IV methylprednisolone, plasma exchange, IV immunoglobulin, and other immune support - is strongly recommended [2]. These treatment options have shown clinical improvement in up to 80% of the patients. The second-line treatment includes the addition of immunosuppressive therapy, such as mycophenolate mofetil or rituximab [3], and was suggested to be immediately started in those who failed to respond or deteriorated during first-line immunotherapy [8]. Because there was not a satisfactory response with two first-line therapies, our patient was started on treatment with IV rituximab whit near-full resolution of psychotic symptoms (decrease of the delusion's systematization and impact), the more prominent and persistent feature in the case.

At present, most patients have a relatively good prognosis after immunotherapy. FDBS can be quickly resolved, and most symptoms can be improved, however, cognitive status is slowly improved, and some patients may have permanent memory impairment [2]. The observed behavior of the patient after discharge with temporary therapeutic drop-out and absence to multiple scheduled consults and repeated attempts to reschedule can evidence some degree of persistent cognitive impairment even if it was not detected on neuropsychological evaluation. If left untreated, most patients progress to permanent hippocampal atrophy with irreversible cognitive dysfunction. A better understanding will be of great significance for an early diagnosis, essentially immunotherapy, and even better prognosis [2]. Because patients can have a negative work-up for malignancies, as occurred in our patient, these patients should be followed up at least over the next five years to search for tumors [9].

The presented case shows that predominantly psychotic symptoms do exist in the context of anti-LGI 1 LE. Patients with anti-LGI 1 LE often present with psychiatric signs, such as behavioral and personality changes, hallucinations, agitation, and sleep disturbances. At an early stage of anti-LGI 1 LE, symptom presentation usually meets the Diagnostic and Statistical Manual of Mental Diseases - fifth edition (DSM-5) criteria for schizophrenia-spectrum diseases, and a DSM-5 diagnosis of schizophrenia is typically assigned in the absence of any other neurological diagnosis [7]. The delay in diagnosis is typical in patients with anti-LGI 1 LE [3]. In this case, if an EEG was not initially performed the patient could be diagnosed with a schizophrenia-spectrum disease, the correct diagnostic would not be done so promptly and appropriate treatment would not be instituted. Eventually, only if there was no response to the antipsychotics would neurological causes be excluded. Therefore, it is important to rule out medical and neurological causes for someone without a history of past psychiatric illness and who presents with sudden changes in behavior [9].

Our report emphasizes that patients with anti-LGI 1 LE, even if they are young (and women) can present predominantly with new onset psychiatric symptoms and first seek psychiatric help. To make an accurate diagnosis it is necessary to combine clinical presentation with supportive auxiliary examinations such as EEG, lumbar puncture, and MRI [7,9]. Above all, it is most important that clinicians be aware of the possible diagnosis of anti-LGI 1 LE when patients present with acute or subacute psychotic symptoms and other manifestations such as memory impairment and sleep disturbances [10], even if they do not show other typical signs of the disease.

## Conclusions

Some predominantly psychotic and cognitive conditions may be caused by milder forms of anti-LGI 1 LE. However, diagnosis is frequently delayed in these cases, when the other typical symptoms are lacking. Sometimes, the patients can even be misdiagnosed and treated as if they suffered from psychiatric disorders. A misdiagnosis leads to a delay in the timing of immunotherapy, predisposing the patient to a poorer prognosis. We should be aware of the diagnosis of anti-LGI-LE when evaluating acute to sub-acute psychiatric illness with cognitive involvement even if patients are young and female and especially if they have no previous significant psychiatric history. The earlier the diagnosis is established, and treatment initiated, the lowest the possibility of long-term sequelae, and with that, the chances of cognitive decline will be frankly diminished, which will allow patients to have a better prognosis overall.
